# 

**Published:** 1845-07

**Authors:** 


### MEDICO-CHIRURGICAL

### REVIEW,

AND

### JOURNAL OF PRACTICAL MEDICINE.

v. [43]

### NEW SERIES.

### VOL. II.

APRIL 1 TO SEPTEMBER 30, 1845.

Surgeon General's Office LIBRARY Washington, D.C.
57483

LONDON:

SAMUEL HIGHLEY, 32, FLEET STREET,

1845.

W1
ME75T

PRINTED BY F. HAYDEN,
Little College Street, Westminster.

### INDEX.

A.

Abdomen, penetrating wound of ... 237
Abscess, hepatic.............. 59, 498
Abscess, pulmonary .............. 112
Abscess, treatment of.............. 157
Abscess, mammary .............. 160
Abscess, metastatic .............. 499
Accidental productions, similarity of 224
Aconite in cardiac disease.......... 176
Acrita, nervous system in.......... 8
Addison, Dr. on pathology of phthisis 196
Addison, Mr. on process of nutrition 148
Agricultural analysis, Mitchell on .. 195
Agriculture, chemistry applied to.... 194
Alcohol, pathological effects of...... 257
Alison on diseases of the heart ...... 249
Ammonia, a source of the nitrogen of plants ........................ 187
Ammonia as a manure . ... ....... 189
Amphioxus lanceolatus, nervous system of ........................ 12
Anasarca in children .............. 99
Anatomy, Arris and Gales' Lectures 155
Anatomy & Physiology, Cyclopædia, 1, 470
Anatomy, Physiological, Todd and Bowman's ................ 1, 470
Anderson on nervous system........ 8
Aneurism, compression in.......... 251
Anger, influence on health ........ 369
Angina, pseudo-membranosa........ 86
Anus, fissure of the .............. 114
Anxiety as a cause of disease ...... 370
Apoplexy, Copeman on............ 103
Apoplexy, treatment. ............ 105
Apoplexy, cases of .......... 107, 206
Apoplexy in children .............. 101
Apoplexy, meningeal.............. 217
Apoplexy, pulmonary ............ 227
Apoplexy, pulmonary, in children .. 101
Apoplexy, not caused by railways.... 552
Arachnida, nervous system of ...... 10
Arachnoid, fluid of the ............ 470
Armies, Jackson on .............. 514
Arsenic, poisoning from, urine in.... 236
Ascites in children................ 98
Auscultation in children .......... 72
Auscultation, Latham on cardiac.... 164
Auscultation in peritonitis ........ 252
Auscultation, cerebral ............ 332

B.

Badeley on railway travelling ...... 552
Baron on accidental productions .... 224
Barthez on diseases of children .. 70, 386
Becquerel on the blood in disease.... 263
Bebeerine, medical properties ...... 254
Bellingham on aneurism .......... 251
Bile, uses of, in the economy ...... 493
Bile, suppression of secretion ...... 507
Biliary ducts, obstruction of........ 505
Binn's anatomy of sleep............ 354
Bird, Dr. G. on diseases of children .. 211
Births, proportion of male to female.. 130
Bischoff on menstruation .......... 525
Blood, coagulation in the veins...... 259
Blood, changes in disease .......... 263
Blood, condition in phthisis ........ 265
Blood, condition in inflammation.... 270
Blood, examination of buffy........ 459
Body, influence of the mind on...... 355
Boismont, Brierre de, on acute delirium 229
Boismont on hallucinations ........ 354
Bouchut on diseases of children.. 70, 386
Bouchut on coagulation of blood .... 259
Boussingault's rural economy ...... 184
Bowman's physiological anatomy.. 1, 470
Brain, inflammation of, in children.. 94
Brain, tubercles in children ........ 421
Brain, size and weight of .......... 476
Brain, convolutions of ............ 476
Brain, Foville on anatomy of........ 479
Brain, relations to cranium ... .... 482
Brain, physiology of ... .......... 487
Brain, seat of sensation and volition 488
Braithwaite's Retrospect . ........ 550
Breast, abscess of ................ 160
Breech, management of presentation, 535
Bronchial glands, tubercles of ...... 413
Bronchitis in children.............. 76
Broussais, doctrines of ............ 119
Bubo, primary, Gibert on.......... 42
Bunion, Durlacher on ............ 242
Burial-clubs, dangers of............ 315
Burnett, Sir W. portrait of ........ 255
Burns, Chelius on ................ 158

C.

Cabanis, Rapports du Physique, &c... 354
Cachexia, coagulation of blood in.... 259

Carbon, deposition in the lungs, 228, 282
Caries, scrofulous ............... 116
Cataract, Mr. C. G. Guthrie on...... 566
Cellar-habitations in towns ........ 308
Cells, agency of, in secretion ...... 28
Cellular tissue, induration of........ 97
Cerebral convolutions, the......... 476
Cerebro-spinal fluid ............. 471
Chancre, Gibert on .............. 41
Chelius' system of surgery.......... 152
Chemistry, influence of on medical doctrines .................... 120
Chemistry, organic, rapid progress of 185
Chemistry, organic, neglect of, in England ....................... 463
Chilblains, cold in ............... 546
Children, diseases of .......... 74, 386
Children, physiological condition of.. 71
Children, examination of sick ...... 72
Children, administration of medicine to.......................... 73
Children, illegitimate, number of.... 130
Children, remittent fever of ........ 211
Children, tuberculization of ........ 406
Children, murder of by burial-clubs, 315
Chlorosis, state of the blood in...... 264
Cholera, relation to fever .......... 553
Cholera, treatment of.............. 554
Chorea, treatment of. ............ 392
Cirrhosis of the liver.............. 496
Classes, working, bad condition of .. 290
Climacteric disease, the............ 371
Cold-water cure, Mayo on.......... 546
Colquhoun on somnambulism ...... 354
Contraction of the extremities in children........................ 393
Convulsions in children............ 389
Corns, Durlacher on .............. 239
Corns, production of.............. 544
Coryza in children................ 85
Cranio-malacia, Widtmann on ...... 272
Cranium, form of, modified by the ventricles ................... 482
Crosse on inversio uteri............ 467
Croup, Rilliet and Barthez on ...... 87
Croup, tracheotomy in ........... 88
Croup, *false*, diagnosis of . ........ 90
Croup, case of .................. 213

D.

Dawson on marriage .............. 245
Deafness, Kitto on............... 250
Death, sudden, Francis on ........ 205
Death, registration of cause of...... 552
Deaths, number in England ........ 132
Delirium, acute, De Boismont on.... 229
Diaphoresis, puerperal ............ 540
Diarrhœa in children.............. 92
Dictionary of medical terms ........ 253
Diphtheritis in children............ 86
Diphtheritis, cutaneous............. 97
Disease, influence of the mind on.... 356
Disease, curative influence of Nature in 381
Disease, influence of temper on .... 383
Diseases, parallelism of ............ 277
Drainage, defective, in towns ...... 293
Drainage, improvements of .... 297, 317
Dropsy in children................ 97
Dropsy after scarlatina ............ 213
Duncan on public health ...... 306, 311
Durlacher on corns .............. 239
Dysentery with expulsion of mucous membrane .................... 111
Dysentery, connexion with cholera .. 553
Dysentery, chronic, treatment of.... 555

E.

Eisenberg on diseases of the foot.... 544
Emotions, effect on sensation ...... 361
Emphysema, pulmonary, in children, 82
Emphysema, case of fatal .......... 275
Empiricism, prevalence of.......... 379
Employments, influence on health .. 309
Empyema, purulent expectoration in, 557
Encephaloid matter, Baron on ...... 227
Encephalon, anatomy of ........... 475
Encephalon, physiology of.......... 487
Endocarditis, Latham on .......... 168
Endocarditis, treatment of ......... 179
Enteritis in children .............. 92
Epilepsy, treatment of ............ 460
Erysipelas in children ............ 96
Experiments, cruel on animals ...... 462
Eye, extirpation of................ 117
Eyre, Sir J., on exhausting diseases.. 67
Fat, Boussingault on ............. 192
Fear, as a cause of disease.......... 367
Fever, isopathic conversion of ...... 278
Fever, connexion of, with cholera and dysentery ..................... 553
Fever from unhealthy localities ..... 313
Fever, *intermittent*, antagonism to phthisis ..................... 449
Fever, intermittent, pneumonia in .. 450
Fever, *puerperal*, nature of ........ 536
Fever, puerperal, from defective ventilation ....................... 322
Fever, *remittent*, of children...... .. 211
Fever, *typhoid*, in children . ........ 392
Fever, *typhus*, Schoenlein on ...... 427
Fever, typhus, calomel in........... 427
Fever, typhus, state of blood in, 430, 442
Fever, typhus, pulmonic affections in, 431
Fever, typhus, relation to intermittent 432
Fever, typhus, bleeding in.......... 434
Fever, typhus, prognosis of ........ 435
Fever, typhus, urine in........436, 441
Fever, typhus, intestinal ulcers in, 537, 459
Fever, typhus, cerebral symptoms .. 438 443—5
Fever, typhus, diarrhœa in ........ 440
Fever, typhus, purgatives in......... 459

Fever, typhus, convalescence of .. 441—4
Fever, typhus, enlarged spleen in, 443, 556
Fever, typhus, eruptions in...... 440—4
Fever, *yellow*, black vomit in ...... 281
Fibrine in inflammation ........... 270
Foot, Eisenberg on diseases of...... 544
Foot, sweating of the............... 546
Foot, neuralgia of the ............ 241
Foville on the nervous system...... 478
Francis on sudden death .......... 205
Frog, spinal nerves in ........... 13

G.

Gall-*bladder and ducts*, diseases of .. 503
Gall-*stones*, formation & symptoms of, 510
Ganglions, synovial ............... 115
Gangrene in children.............. 102
Gangrene, Chelius on .............. 158
Gangrene, dissemination of ........ 507
Gastro-enterite of children......... 90
Gibert on diseases of the skin . .... 35
Gintrac on intra-thoracic tumors.... 273
Glands, Simon on secretion by...... 28
Glands without ducts.............. 31
Glands, lymphatic ................ 124
Glottis, œdema of............ 219, 435
Gonorrhœa, Ricord on ............. 549
Gonorrhœa of the nose ............ 161
Gonorrhœa, with secondary symptoms 42
Goodsir's anatomical and pathological observations ................... 121
Grief, effects of, on health........... 373
Gums, lancing of ................. 142
Guthrie, Mr. C. G., on cataract..... 566
Guy's Hospital Reports............ 196

H.

Hæmatemesis in children .......... 101
Hæmoptysis, oxide of silver in...... 68
Hæmoptysis in children ........... 101
Hæmorrhages in children .......... 101
Hæmorrhoids, treatment of ........ 115
Hairs acting as foreign bodies ...... 113
Hallucinations, De Boismont on .... 354
Harden on parallelism of disease .... 277
Hastings on Pulmonary Consumption 243
Headache, sitz-bath in ............. 548
Health, public, defective condition of, 138
Health, public, defective condition of, 290
Health, medical officers of ......... 318
Heart, diseases of, Latham on ...... 163
Heart, auscultation of ............. 165
Heart, Furnivall on ............... 175
Heart, hypertrophy and dilatation of, 180
Heart, valvular disease of.......... 181
Heart, disease of, as a cause of sudden death ........................ 207
Heart, rupture of ................. 209
Heart, steel in diseases of the ...... 249
Heart, new analysis of sounds of.... 562
Hepatitis, Budd on................. 495
Hepatitis in children .............. 94
Hernia, traumatic omental......... 237
Hidrosis, puerperal ............... 540
Hocken on Retinitis .............. 464
Hood on Diseases of Children ...... 70
Hope, influence of in disease........ 365
Hydatids of the liver.............. 513
Hydatids of the lungs ............. 227
Hydrocephalus, Rilliet and Barthez on 99
Hydrocephalus, Smith on.......... 326
Hydrocephalus, stages of .......... 329
Hydrocephalus, pulse in ........... 330
Hydrocephalus, diagnosis of........ 331
Hydrocephalus, auscultation in...... 332
Hydrocephalus, etiology of ......... 333
Hydrocephalus, morbid anatomy of.. 334
Hydrocephalus, treatment of........ 335
Hydropathy, Soltau on............. 250
Hydrothorax in children ........ .. 98
Hygiene, public, defects in ....138, 290

I.

India, Webb on diseases of ........ 49
Infants, induration of cellular tissue of 97
Infants, syphilis in................ 47
Inflammation in children .......... 75
Inflammation, state of blood in..263, 270
Insane, acute delirium of the........ 229
Insanity, hereditariness of ......... 223
Insanity, emetics in .............. 249
Insanity, phys. causes of, exaggerated 538
Intestinal irritation, puerperal ...... 538
Intestinal villi, Goodsir on ........ 123
Intestines, inflammation of, in children 92
Intestines, tuberculization of, in children........................ 419
Intestines, *large*, detachment of mucous membrane from............ 111
Iodine in syphilis ................. 276
Isopathia, Harden on.............. 277

J.

Jackson, Dr. biography of.......... 515
Jaundice from suppressed bile ...... 507
Jaws, O'Shaugnessy on diseases of .. 147
Jaws, abscess of.................. 148

K.

Kidney, tuberculization in children .. 420
Kitto, the Lost Senses ............ 250
Knox on Irish Watering Places ..... 551

L.

Labour, relaxation of ligaments in ... 529
Labours, classification of .......... 533
Lanceolet, nervous system of ....... 12
Larrey, Hipp. on Extrusion of the Omentum ..................... 237
Laryngitis, spasmodic ............. 90
Larynx, tubercles in children ...... 417
Latham on Diseases of the Heart.... 163

Leeches, re-application of ......... 118
Leeches, diseases of .............. 119
Leg, singular case of fracture of .... 117
Leucorrhœa, uterine .............. 549
Life, duration of, in England........ 311
Liver, abscess of ................ 59
Liver, fatty .................257, 509
Liver, fatty, in children............ 420
Liver, Budd on diseases of.......... 493
Liver, structure and functions of.... 493
Liver, congestion of .............. 494
Liver, adhesive inflammation of..... 495
Liver, cirrhosis of ............... 496
Liver, condition during obstruction of the ducts...................... 505
Liver, gangrene of................ 507
Liver, cancer of.................. 511
Liver, hydatids of ............... 513
Liver, tubercle of, in children ...... 420
Liverpool, defective sanitary condition of ....................... 307, 313
Login on field carriage ............ 557
Lungs, acute abscess of............ 112
Lungs, hydatids................... 227
Lungs, decomp. of carbon in....228, 282
Lungs, œdema of, in children ....... 98
Lungs, gangrene of, in children .... 102
Lungs, tubercle of, in children...... 415

M.

Macilwain on tumors............... 422
Maclagan on bebeerine ............ 254
Maculæ syphiliticæ ............... 46
Manures, Boussingault on.......... 188
Marriages, number in England...... 129
Martin on Public Hygiene.......... 128
Martin, Mr. J. R., testimonial to.... 569
Mason on Female Health .......... 246
Mayo on the Cold-water Cure ...... 546
Measles, complications of.......... 402
Measles, prognosis................ 405
Measles, incubation .............. 406
Medical reform ................... 256
Medical science, Cowan on ........ 457
Medicines, administration to children, 73
Melanosis, Baron on .............. 220
Memoires de l'Academie de Medicine, 215
Menstruation, recent theory of...... 525
Menstruation, nature of discharge in, 528
Mesenteric phthisis in children...... 418
Midwifery, present state of ........ 523
Mind, influence of on the body...... 355
Mitchell, agricultural analysis ...... 195
Moore on Powers of the Soul ....... 354
Moreau's System of Midwifery..... 523
Mortality of Towns ...... 137, 307, 311
Mortality, ratio of, in England.. 132, 311
Mortality, according to hygienic condition .................... 312, 314
Mortification, acute .............. 158
Mortimer on the Teeth ............ 141
Mouth, gangrene of, in children .... 102
Muguet, Bouchut on ............. 86
Myelitis mistaken for rheumatism .. 452
Myriapoda, nervous system of....... 10

N.

Nails, diseases of the.............. 243
Neison on influence of employments, 309
Nephritis in children .............. 94
Neuroses of children .............. 387
Nerve, spinal accessory............ 492
Nerves, origins and terminations of.. 6
Nerves, *spinal*, origin of ........... 474
Nervous system, anatomy of...... 2, 470
Nervous system, comparative anatomy 7
Nervous system, physiology of...... 483
Nervous *matter*, analysis of ........ 4
Nervous matter, the grey........ 4, 484
Nervous matter, fibrous ........... 5
Nervous power distinguished from electricity ...................... 485
Newport on nervous system ........ 10
Nipple, syphilis of................ 45
Nitrates, the, as manures .......... 190
Nitrogen, assimilation by plants .... 186
Nose, gonorrhœa of .............. 161
Numerical method ........... 120, 458
Nutrition, cell-theory of .......... 122
Nutrition, Addison on ............ 149

O.

Occiput, soft, Widtmann on........ 272
Omentum, extrusion of............ 237
Ovary, agency in menstruation ..... 525
Ovariotomy, Cowan on............ 460
Ovum, structure of ............. 530
Oxygen, Riadore on influence of .... 247

P.

Pacinian corpuscles .............. 17
Palmer's Dictionary of Medical Terms 252
Panaris, epidemic ................ 115
Paracentesis thoracis.............. 455
Paraplegia, simulated.............. 559
Parchappe on the sounds of the heart, 565
Paving, defective, in towns ........ 300
Pellagra in France ................ 285
Pelvis, relaxation of ligaments during labour ........................ 529
Pelvis, Carus' circle of the ....... 529
Pericarditis, Latham on............ 169
Pericarditis, treatment of .......... 179
Pericarditis in children ............ 85
Peritonitis, friction vibrations in .... 253
Peritonitis, puerperal.............. 536
Peritonitis, puerperal, false ........ 539
Peritonitis in children ............ 93
Peritonitis, tubercular ............ 418
Pertussis, Rilliet and Barthez on.... 387
Pertussis, alum in ................ 215
Pharynx, gangrene of, in children.... 103

Phlegmasia dolens ................ 535
Phlegmasiæ in children ... ......... 75
Phlegmasiæ, changes of the blood in the .................... 263, 270
Phthisis, condition of the blood in .. 265
Phthisis, fatty liver in.. .. 266, 257, 509
Phthisis in the lower animals ...... 267
Phthisis, bronchial................. 413
Phthisis, pulmonary, in children .... 415
Phthisis, pleural................... 417
Phthisis, laryngeal ................ 417
Phthisis, peritoneal ............... 418
Phthisis, mesenteric ............... 418
Phthisis in antagonism with ague.... 449
Phthisis, naphtha in .......... 243, 460
Phthisis, Addison on the Pathology of 196
Phthisis, paracentesis in............. 244
Phthisis, influence of employments in producing ..................... 309
Physiology, abstract pursuit of...... 462
Placenta, structure & function of, 124, 530
Playfair on Health of Towns ...... 308
Pleura, tuberculization of.......... 417
Pleurisy in children ............... 83
Peurisy, Schoenlein on.............. 452
Pleurisy, complicated with spinitis .. 451
Pleurisy, effusion from, treated...... 454
Pneumonia in children ............ 78
Pneumonia, Schoenlein on ........ 445
Pneumonia, coexistence with carditis 447
Pneumonia, value of auscultation in 448
Pneumonia, complicated with ague .. 450
Pneumonia, treatment of .......... 451
Pneumo-thorax in children ........ 417
Population, density of ............ 307
Pregnancy, signs of .............. 532
Pregnancy, menstruation during .... 532
Pregnancy, *extra-uterine*, case of.... 235
Presentation, vertex, varieties of.... 533
Prus on Meningeal Apoplexy ...... 217
Prussic acid, poisoning by.......... 201
Puerperal fever .................. 536
Pulse in children ................ 71
Purpura in children ............... 102
Pyrosis, oxide of silver in .......... 67

Q.

Quackery, Cowan on............... 461

R.

Raciborski, de la puberté, &c. ....... 523
Ramsbotham's obstetric medicine.... 523
Ramsbotham on puerperal fever .... 53
Ranking's Half Yearly Abstract .... 550
Rayer on phthisis in animals........ 267
Registrar-General, Sixth Report of .. 128
Religious enthusiasm, effects of .... 375
Retinitis, Hocken on............... 464
Rheumatism, diseases of heart in .. 171—5
Rheumatism, treatment ........176—8
Rheumatism in children .. ......... 95
Riadore on influence of oxygen...... 247
Rilliet on diseases of children .. 70, 386
Robertson on Sexual diseases ...... 548
Roseola, syphilitic ............... 43
Rouanet's analysis of sounds of heart, 562
Rural economy, Boussingault's...... 184
Rupia, syphilitic ............... 43

S.

Sanitary bill, provisions of ........ 317
Scabies, Gibert on ................ 47
Scalp, disease of, repulsion of ...... 96
Scarlatina, anasarca after...... 100, 213
Scarlatina, cases in 1842 .......... 133
Scarlatina, puerperal.. ..... ...... 543
Scavenging, defective ............ 301
Schoenlein's Clinical Reports ...... 425
Secretion, cell-theory of .... 26, 32, 124
Segalas on uretro-plastie .......... 216
Sensations, effect of emotions on.... 361
Sewerage, defective.......... 293, 317
Sexes, proportion of births ........ 130
O'Shaugnessy on diseases of the jaws, 147
Silver, oxide, employment of.... 67, 286
Simon on the thymus gland ........ 18
Skin, Gibert on diseases of.......... 35
Skin diseases, causes of ........... 36
Skin diseases, metastasis and crisis of, 38
Skin diseases, treatment of ....... 38
Skin diseases, classification of ...... 41
Skin diseases, syphilitic............ 43
Skin diseases, repercussion of ...... 96
Skin, gangrene of, in children ...... 103
Small-pox, post-mortem appearances 396
Small-pox, complications .......... 398
Small-pox, management of pustules in 400
Small-pox, prevalence in England, 133, 337
Small-pox, inoculation of, improper.. 342
Small-pox in India................ 346
Small-pox in the cow ............ 349
Smith on hydrocephalus .......... 326
Soltau on hydropathy.............. 250
Soul, Moore on the power of the.... 354
Sounds of the heart, M. Parchappe on 565
South's translation of Chelius ...... 152
Spleen, tuberculization of.......... 420
Spleen in fever .......... 57, 443, 556
Spleen, uses of the................ 50
Spleen diseases in India........... 52
Spleen, abscess of ................ 56
Spleen, uses in disease ............ 556
Spinal cord, anatomy of .......... 473
Spinal cord, Foville on .......... 478
Spinal cord, functions of .......... 485
Spinal cord, Valentin on reflex theory of........................ 487
Spinal cord, influence on volition and sensation................ ...... 489
Spittal on vibrations in peritonitis .. 252
Stewart on small-pox in India ...... 346
Stilling on the nervous system ...... 2

Stomach, diseases of, in children .... 90
Stomach, softening of ........ 90, 275
Stomatitis in children ............ 65
Streets, defective cleansing of ...... 301
Study, excessive effects of .......... 359
Suicide, hereditary disposition to.... 224
Surgery, Chelius' system of ........ 152
Syphilides, the various ............ 43
Syphilis, treatment ........... 276, 548
Syphilis in the infant........... 47, 213
Syphilis of the nipple .............. 45
Syphilis of the skin .............. 43

T.

Taylor on poisoning by Prussic acid.. 201
Teeth, Mortimer on the............. 141
Teeth, early cleansing of ........ 143—5
Teeth, removal of, in children.... 143—4
Teeth, filing and scaling of ........ 146
Testimonial to J. R. Martin, Esq..... 569
Thymus gland, Simon on the........ 18
Thymus gland, various opinions on the 19
Thymus gland, development ........ 21
Thymus gland, structure and secretion 23
Thymus gland, comparative anatomy 25
Thymus gland, morphology ........ 26
Thymus gland, functions of ........ 34
Tierney on variola vaccinia ........ 337
Toes, neuralgia of ................. 241
Toogood's hints to mothers ........ 247
Tooth-powders, Mortimer on ...... 146
Torpedo, nervous system of........ 14
Towns, defective hygiene of.... 293, 138
Towns, density of population of .... 307
Towns, ratio of mortality in .... 311—13
Transactions of the Provincial Association..................... 456
Tuberculization in children ........ 406
Tuberculization, pathological anatomy 407
Tuberculization of different organs 408, 421
Tuberculization, symptoms of ...... 409
Tuberculization, causes of......... 410
Tuberculization, influence of prior disease on ..................... 411
Tuberculization, treatment of ...... 412
Tumours, intra-thoracic .......... 273
Tumours, Macilwain on .......... 422
Tympanitis, puerperal ........... 538

U.

Uretro-plastie, Segalas on.......... 216
Urine in disease......... 330, 436, 441
Uterus, inversion of .............. 467
Uterus during menstruation........ 528
Uterus, puerperal congestion of .... 535

V

Vaccination in India .............. 350
Vaccination in Sweden............ 338
Vaccination, amount of protection of .................... 339, 344
Vaccination and *re-vaccination*.. 342, 345
Vaccination, French Report on.. 269, 343
Vaccination compulsory .......... 352
Vaccination, influence of, on the non-vaccinated ................... 399
Valleix on œdema glottidis ........ 219
Varioloid, Serres on the .......... 344
Veins, coagulation of blood in the .. 259
Ventilation, neglect of ........ 308, 322
Vertex, varieties of presentation of .. 533
Vomit, the black, nature of ........ 281

W

Warts, treatment of.......... 243, 546
Water, defective supplies of........ 303
Whitlow, epidemic .............. 115

### BIBLIOGRAPHICAL RECORD.

1. A Collection of Cases of Apoplexy, with an Explanatory Introduction. By EDWARD COPEMAN, Surgeon. 8vo, pp. 211. Churchill. 1845.

2. The Diagnosis, Prevention, and Treatment of Diseases of the Heart and of Aneurism, with Observations on Rheumatism. By J. J. FURNIVALL, M.D. 8vo, pp. 224. London, 1845.

3. The general Nature and Treatment of Tumours. By GEORGE MACILWAIN. 8vo, pp. 231. Churchill, 1845.

4. On the Remedial Influence of Oxygen or Vital Air, Nitrous Oxide, and other Gases, Electricity and Galvanism. By J. EVANS RIADORE, M.D., F.L.S. 8vo, pp. 185. Churchill, 1845.

5. Observations on the Growth and Irregularities of Children's Teeth, followed by Remarks and Advice on the Teeth in general; to which is added, a short Essay on Artificial Teeth. By W. H. MORTIMER. 12mo, pp. 141. London, 1845. Highley.

6. The Structure of the Lungs, anatomically and physiologically considered with a view to exemplify the Wisdom, Power, and Goodness of God, as revealed and declared in Holy Writ. The Warnford Prize Essay for the Year 1844. By JOHN MOORE.

7. A System of Surgery. By J. M. CHELIUS. Translated from the German, with Notes and Observations, by J. F. SOUTH, Surgeon to St. Thomas's Hospital. 8vo, Parts I. to III. Renshaw, 1845.

8. A Physiological Essay on the Thymus Gland. By JOHN SIMON, F.R.S. 4to, Plates, pp. 116. London, Renshaw, 1845.

9. The Power of the Soul over the Body considered in Relation to Health and Morals. By GEORGE MOORE, M.D. Pp. 313. Longman & Co. 1845.

10. Practical Observations on the Diseases most fatal to Children; with reference to the Propriety of treating them as proceeding from Irritation, and not from Inflammation. By P. HOOD. Post 8vo, pp. 231. Churchill, 1845.

11. On Diseases of the Jaws, with a brief Outline of their Surgical Anatomy, and a Description of the Operations for their Extirpation and Amputation, with Cases and Illustrations By RICH. O'SHAUGHNESSY. 8vo. pp. 100. Calcutta, 1844.

12. Observations on the Education and Examinations for Degrees of Medicine, as affected by the New Medical Bill, in a Letter to the Right Hon. Sir James Graham, Bart. By RICHARD QUAIN, F.R.S. 8vo, pp. 60. Murray, 1845.

13. Contributions to the Medical History and Treatment of Sexual Diseases. By JOHN H. ROBERTSON, M. D. 8vo, pp. 80. Glasgow, 1845.

14. Glanders and Farcy in the Horse. By WILLIAM PERCIVAL, M.R.C.S. Being the second and concluding Part of Vol. III. of the Author's Hippopathology. 8vo. pp. 199. Longman & Co. 1845.

15. The London Medical Directory for 1845: containing the Name, Address, Qualifications, Official Appointments, and Literary Productions of every Physician, Surgeon, and General Practitioner resident in London. 12mo, pp. 188. Churchill.

16. The British American Journal of Medical and Physical Sciences. Edited by ARCHIBALD HALL, M.D., Edin. Montreal, 1845.

17. A Practical Treatise on the Special Diseases of the Skin, with Cases and numerous Notes collected from the best Authors. By C. M. GIBERT, Physician to the Hôpital St. Louis, &c. Second edition, enlarged. Translated by EDGAR SHEPPARD. 12mo, pp. 362. Churchill, 1845.

18. Manual of Agricultural Analysis. By JOHN MITCHELL. Small 8vo, pp. 143. London, 1845. Simpkin & Marshall.

19. The Physiological Anatomy and Physiology of Man. By R. B. TODD, M.D., F.R.S., and W. BOWMAN, F.R.S. Part II. 8vo, pp. 248. Parker, 1845.

20. Pulmonary Consumption successfully treated with *Naphtha*, with Cases from other Medical Men in support of that Treatment. Second edition, revised and enlarged. By JOHN HASTINGS, M.D. 8vo, pp. 264. London, 1845.

21. A few Remarks addressed to the Public on Hydropathy, or the Cold Water Cure, and other Panaceas. By W. F. SOL-

TAU, M.B. 8vo, pp. 72. Hamilton, London, 1845.

22. Hints to Mothers on the Management of Females at the Age of Puberty. By JONATHAN TOOGOOD. 8vo, pp. 20. 1845.

23. Transactions of the Provincial Medical and Surgical Association. New Series. Vol. I. 8vo, pp. 426. Churchill, London. 1845.

24. Observations on Friction Vibrations in Peritonitis. By ROB. SPITTAL, M.D., F.R.S. E. 8vo, pp. 20. 1845.

25. Annual Report of the Medical College of Bengal. Session 1844-5.

26. Observations on the Employment of Compression in Aneurism. By O'B. BELLINGHAM, M D. 8vo, pp. 14. Dublin, Hodges and Smith. 1845.

27. Vital Chemistry.—Lectures on Animal Heat. By THOMAS SPENCER, M.D. 8vo, pp. 114. Geneva, N. Y. 1845.

28. Outlines of Chemistry, for the Use of Students. By WM. GREGORY, M.D. Part II. Organic Chemistry. 12mo, pp. 588. London, Taylor & Walton. 1845.

29. American Journal of the Medical Sciences. Edited by ISAAC HAYS, M.D. 8vo, pp. 532. Philadelphia, Lea and Blanchard. 1845.

30. Sixth Annual Report of the Registrar-General of Births, Deaths, and Marriages in England. 8vo, pp. 666. London. 1845.

31. Report of the Sanatory Condition of Nottingham, Coventry, Leicester, Derby, Norwich, and Portsmouth. By JAMES R. MARTIN, Esq. Folio, pp. 58.

32. The Veterinarian, Nos. for January to June, 1845.

33. Annual Report of the Managers of the State Lunatic Asylum, 1845. Albany. 8vo, pp. 53.

34. The Sanatory Condition of the Laboring Population of New York. By JOHN H. GRISCOM, M.D. 8vo, pp. 58. New York, 1845.

35. Remarks on Physicians, Surgeons, Druggists, and Quacks. By Surgeon SNIPE. 8vo, pp. 65. London, Highley. 1845.

36. Chemistry, Meteorology, and the Function of Digestion, considered with reference to Natural Theology. By WILLIAM PROUT, M.D. F.R.S. 8vo, pp. 515. London, Churchill. 1845.

37. The Irish Watering Places, their Climate, Scenery, and Accommodations, including Analyses of the Principal Mineral Springs. By Dr. R. KANE; and Remarks on the various Forms of Disease to which they are adapted by ALEX. KNOX, M.D. Small 8vo, pp. 332. Dublin, Curry & Co. 1845.

38. Spinal Affections and the Prone System of treating them. By JAMES COLES, Surgeon. 12mo, pp. 320. London, Houlston & Co. 1845.

39. Seven Lectures on Somnambulism. Translated from the German of Dr. ARNOLD WIENHOLT. By J. C. COLQUHOUN. 12mo, pp. 219. Edinb. Black & Co. 1845.

40. Prize Chemical Report of Surgical Cases treated at the Queen's Hospital, Birmingham. By JOHN MOORE. 8vo, pp. 65. Churchill, 1845.

41. Practical Notes on Insanity. By J. B. STEWARD, M.D. 12mo, pp. 122. London, 1845.

42. The Quarterly Medical and Surgical Journal for the North-western Provinces. Nos. 1, 2, 3, & 4, for 1844. Delhi, 1845.

We hail with pleasure this new accession to periodical literature, edited and published in the centre of India! In our next number we intend to offer copious extracts from the work. We shall be happy toexchange Journals through the medium mentioned.

43. Pathologia Indica, or the Anatomy of Indian Diseases, Medical and Surgical, based upon Morbid Specimens from all Parts of India in the Museum of the Calcutta Medical College. Illustrated by Cases, &c., and Comments, Physiological, Practical, and Historical. By ALLAN WEBB, B.M.S., &c. Part I. Calcutta, Cary & Co. 1844.

44. Transactions of the Medical and Physical Society of Bombay. Part VI. Bombay, 1843.

45. Des Hallucinations, ou Histoire raisonnée des Apparitions, Visions, Songes, de l'Extase, du Magnetisme et du Somnambulisme. Par A. BRIERRE de Boismont. 8vo, pp. 615. Paris, 1845. Bailliere.

46. Lectures on Subjects connected with Clinical Medicine, comprising Diseases of the Heart. By P. M. LATHAM, M.D., Physician Extraordinary to the Queen, &c. Vol. I. pp. 374. London, 1845. Longman & Co.

47. On Cataract and its appropriate Treatment. By CHARLES G. GUTHRIE. 8vo, pp. 127. London, 1845.

